# Evaluation of the Influence of the Combination of pH, Chloride, and Sulfate on the Corrosion Behavior of Pipeline Steel in Soil Using Response Surface Methodology

**DOI:** 10.3390/ma14216596

**Published:** 2021-11-02

**Authors:** Nguyen Thuy Chung, Yoon-Sik So, Woo-Cheol Kim, Jung-Gu Kim

**Affiliations:** 1 School of Advanced Materials Science & Engineering, Sungkyunkwan University, 300 Chunchun-Dong, Jangan-Gu, Suwon 440-746, Korea; chung.ngthuy@g.skku.edu (N.T.C.); soy2871@gmail.com (Y.-S.S.); 2 Technical Efficiency Research Team, Korea District Heating Corporation, 92 Gigok-ro, Yongin 06340, Korea; Kwx7777@kdhc.co.kr

**Keywords:** response surface methodology, Box–Behnken design, modeling, soil environment, carbon steel corrosion

## Abstract

External damage to buried pipelines is mainly caused by corrosive components in soil solution. The reality that numerous agents are present in the corrosive environment simultaneously makes it troublesome to study. To solve that issue, this study aims to determine the influence of the combination of pH, chloride, and sulfate by using a statistical method according to the design of experiment (DOE). Response surface methodology (RSM) using the Box–Behnken design (BBD) was selected and applied to the design matrix for those three factors. The input corrosion current density was evaluated by electrochemical tests under variable conditions given in the design matrix. The output of this method is an equation that calculates the corrosion current density as a function of pH, chloride, and sulfate concentration. The level of influence of each factor on the corrosion current density was investigated and response surface plots, contour plots of each factor were created in this study.

## 1. Introduction

Buried pipeline systems are a key part of global infrastructure. Any considerable disturbance to the performance of these systems frequently has negative consequences for regional businesses, economies, and citizen living circumstances [1]. Due to the complexity of the medium and surroundings such as soil components, corrosion is one of the leading reasons for pipeline degradation and failure [2]. Therefore, it is necessary to thoroughly understand the processes as well as a comprehension of the cause of external corrosion steel pipelines in soil [3].

The factors influencing soil corrosion include soil components, moisture content, temperature, resistivity, differential aeration, soil type, permeability, and the presence of sulfate-reducing bacteria [4,5,6,7,8]. Due to the complexities of its environment, soil corrosion is not easy to study. A large number of studies on soil corrosion are necessary for the future. This article focuses on the influence of the chemical component, and three corrosive agents selected are pH, chloride, sulfate concentration.

Several authors have previously studied the effects of pH, chloride, and sulfates in synthetic soil and particle soil [9,10,11,12]. However, to our knowledge, no studies have examined the combined influence of these three factors. To study the combined effects of these parameters, different combinations were investigated using statistically designed experiments. However, when using a statistical synthesis method, the number of experiments can be very large. Finding an optimal statistical method is essential. Recently, two widely applied methods are Artificial Neural Networks (ANNs) and response surface methodology (RSM) [13,14,15] and response surface methodology (RSM) has been selected and used herein.

The main objective of this study was to enhance a clear understanding of the effects of the soil environment, especially the influencing factors, on the corrosion of steel pipelines. RSM, based on the design of experiments (DOE), is a set of statistical and mathematical tools for experimental design and optimization of the effect of process variables [16,17,18]. RSM reduces the number of trials and recognizes the influence of component parameters on corrosion current density. To obtain the input statistics of corrosion current density from RSM, electrochemical tests were carried out to formulate an equation that calculates the corrosion current density using the parameters of pH, chloride concentration, and sulfate concentration. After that, the equation was confirmed to be reliable by analysis of variance (ANOVA), and the level of influence of each factor on corrosion current density was investigated by T-test. Finally, to determine the interactions between the corrosive agents, surface contour plots of each factor were created.

## 2. Investigation Scheme Design of Experiment

**Figure 1:**
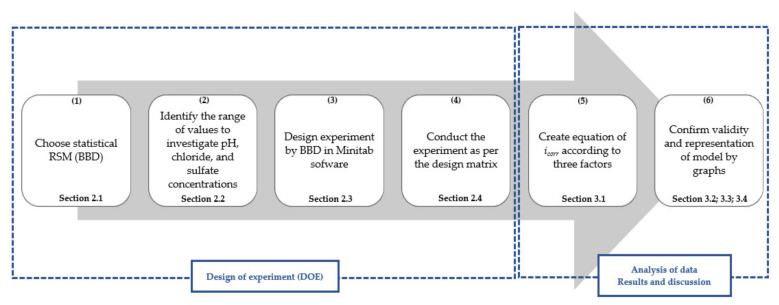
Six steps were implemented to investigate the influence of pH, chloride concentration, and sulfate concentration on the corrosion current density of carbon steel in a soil environment.

To achieve the desired aim, the investigation was planned in the sequence shown in Figure 1.

The above steps will be explained in detail in the following sections.

### 2.1. Response Surface Methodology and Box–Behnken Design

To investigate the influence of several factors on a response, a conventional statistical method can be very useful. However, this approach requires a large number of combinations of different parameter values to determine the effects of those combinations. For example, in this study using the three factors of pH, chloride, and sulfate to obtain the corrosion current density, the maximum, median, and minimum were determined for each factor; therefore, the number of experiments would be at least 3 × 3 × 3 = 27 experiments and thus time-consuming. To simplify the procedure, RSM, a multivariate statistical tool that offers a modern approach to investigating the combined variables, was implemented because it has several advantages [19,20]. Firstly, RSM provides accuracy and process optimization with a good perspective for predictive model innovation; secondly, response surfaces are graphical representations used to illustrate the interactive effects of factors effects on response; and most importantly, a limited number of experiment runs are designed, in comparison to a conventional approach for the same number of estimated factors [20]. RSM is useful for analyzing the effects of parameters and their interactions with each other.

Estimation of coefficients, prediction of responses, and confirmation of the adequacy of the model are process optimization. The response is represented by Equation (1) [19],

*Y = f (X*_1_*, X*_2_*, …,X*_n_) ± *E*, (1)

Here, *Y* is the response, *f* is the response function, *X*_1_… *X*_n_ are the variables of action called factors, *n* is the number of variables, and *E* is the experimental error. These mathematical models are typically polynomials with an unknown structure; however, to fully present linear interactions and quadratic effect, it is preferable to employ a second-order polynomial model [21]. Experiment design could have a significant effect on the accuracy of approximation as well as the cost of building the response surface [22]. In this study, *X*_1_, *X*_2_, and *X*_3_ are the three experimental variables studied—that is, pH, chloride concentration, and sulfate concentration, respectively. The response (*i*_corr_) was related to the selected variables by the second-order polynomial regression model given in Equation (2) [19],

(2) $$Y=\beta_{0}+\sum_{i=1}^{n}\beta_{i}X_{i}+\sum_{i=1}^{n}\beta_{ii}X_{i}^{2}+\sum_{i=1}^{n-1}\sum_{j>i}^{n}\beta_{ij}X_{i}X_{j}+E,$$

Here, $\beta_{0}$ represents the intercept or regression coefficient; $\beta_{i}$,$\;\beta_{ii}$, and $\beta_{ij}$ represent the linear, quadratic, and interaction parameters, respectively; $X_{i}$ and $X_{j}$ are the coded values of the process variables.

Different designs can be used in statistical experiments; the variation between these designs is determined by the number of runs (experiments) and the experiment points chosen. Central composite design (CCD) and Box–Behnken design (BBD) are two extremely helpful and common for fitting a second-order model. Simple factorial or fractional designs are used to create both designs. Simple factorial or fractional factorial designs are used to create both designs. Both designs are built up from simple factorial or fractional factorial designs. BBD is a spherical, three-level fractional factorial design including a center point and middle point of edges of a circle circumscribed on a sphere while CCD is a fractional factorial design. The advantage of BBD is that it just requires a reasonable group of factors to determine the complex response function and voids experiments performed under extreme conditions, and BBD was selected and applied to analyze the influence of combination factors on the response [23]. Response surface methodology has recently been a widespread approach to optimize and analyze diverse products because of the availability of various computer software programs, such as Design Expert, Statistica, MATLAB, and Minitab, which make the application of RSM easier.

### 2.2. Identifying the Range of the Values to Investigate and Preparing Reagents

Before designing the experiment, the effective factors of a system and the range of these factors must be identified. In this study, three factors (pH, chloride, and sulfate) were used and the tested range of these components was determined based on a real soil environment in Korea (pH: 6.8; CaCl_2_: 133.2 ppm; MgSO_4_.7H_2_O: 59.0 ppm; NaHCO_3_: 208.0 ppm; H_2_SO_4_: 48 ppm; HNO_3_: 21.8 ppm) [24]. Table 1 lists the initial concentration of the solution and range of the factors that influence corrosion.

### 2.3. Design of Experiment and Design Matrix

**Figure 2:**
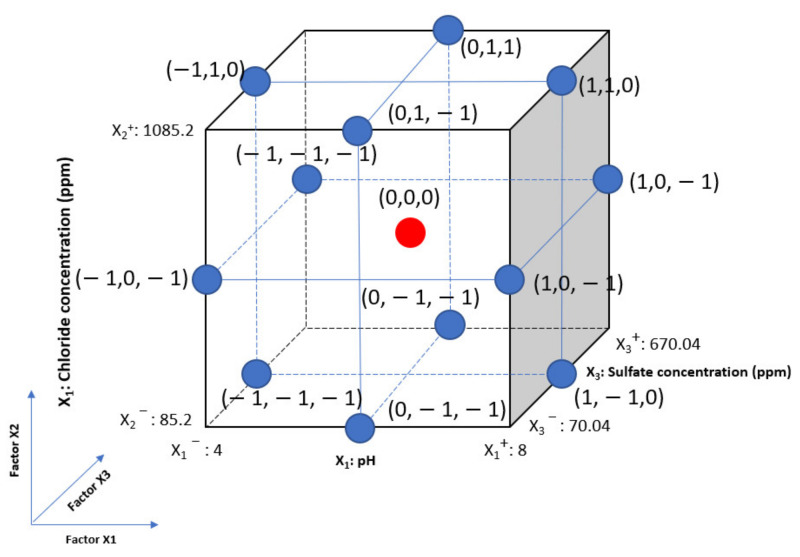
Cube plot design of experiments for corrosion current density according to pH, chloride, and sulfate concentrations by BBD.

Based on the testing range, the design of the experiment was carried out using the Minitab 19 software. For the RSM using Box–Behnken design, an experiment number of *N = k^2^ + k + c_p_* was required, where *k* is the factor number and *c_p_* is experiment runs at the center point. It can be viewed as a cube in Figure 2, which consists of a central point and middle points on the edges. For the statistical calculations, the variables were coded as the minimum, central, and maximum levels of each variable, designated as −1, 0, and 1, respectively, as shown in Table 2. Two ways to collect enough data is to use replicates and add just a few center points. Replicating could be time-consuming; therefore, this design includes 3 experiment runs at the center point. In this study, the number of factors, *k*, was 3 and *c_p_* was 3 times the number of central points (red point in Figure 2); therefore, the total number of experiments was 15 at three levels, as shown in Table 3. This number was significantly reduced compared with the conventional methods.

The results were input into the Minitab 19 software for further analysis. On examining the fit summary, the quadratic model was found to be statistically significant for the response.

### 2.4. Conducting Electrochemical Tests

Next, electrochemical tests were carried out using a three-electrode system configuration based on the matrix values described previously. The experiment was performed using a corrosion cell in which carbon steel SPW400 (0.04% wt. S; 0.04% wt. P; 0.25% wt. C; Bal. Fe—Korean standard) with a 2 mm thickness (a common material for buried pipelines) was used as the working electrode along with a reference electrode (saturated calomel electrode; RE, Qrins), and a counter electrode (two pure graphites; CE, Qrins, Seoul, Korea) [25]. The specimen was polished with SiC paper from 200 to 600 grit sizes, the surface was coated with silicone paste, leaving an exposed area of 1 cm^2^. NaOH and HNO_3_ (Samchun, Pyeongtaek-si, Korea) were used to adjust the pH values, and NaCl and Na_2_SO_4_ (Samchun, Pyeongtaek-si, Korea) were used to adjust chloride and sulfate concentrations, respectively. An open-circuit potential (OCP) was established within 3 h before the electrochemical tests. Tafel plots were obtained by potentiodynamic polarization tests. To prevent mutual polarization, the potentiodynamic polarization tests were separated into anodic and cathodic polarization. The potential range of the cathodic polarization was from 0 V vs. OCP to −0.25 V vs. OCP, whereas that for the anodic polarization was from 0 vs. OCP to 0.5 V vs. OCP, both with scan rates of 0.166 mV/s. Each experiment was carried out with the maintained process variable conditions given in the design matrix.

## 3. Results and Discussion

### 3.1. Electrochemical Test, Second-Order Polynomial Equation, and Statistical Analysis

**Figure 3:**
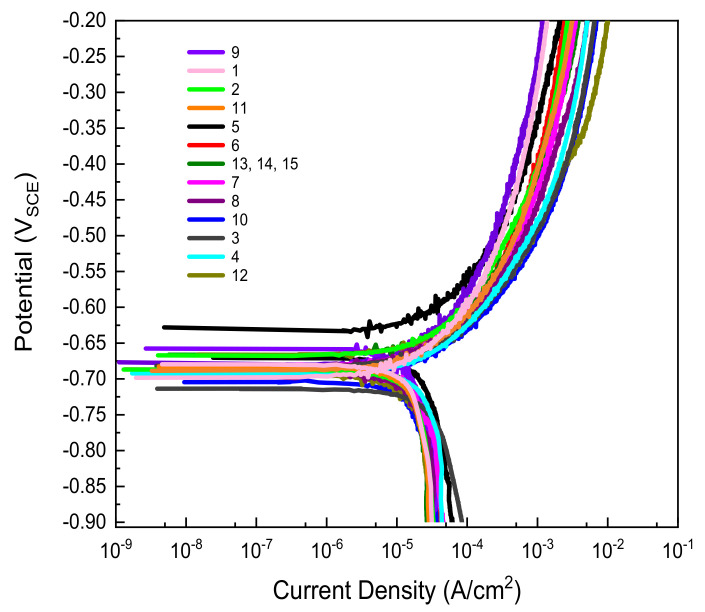
Potentiodynamic polarization curves of carbon steel with variations in pH, chloride, and sulfate concentrations based on an experimental design of RSM with BBD.

Figure 3 shows the potentiodynamic polarization curves used to obtain the corrosion current density for the RSM with BBD. The corrosion current density was calculated using the Tafel extrapolation method.

The results of the corrosion current density for the RSM experiment design are displayed in Table 4; the collected data results were inserted into the DOE. For convenient comparison, the order of corrosion current density increases from the minimum value to the maximum value. Based on the results shown in Table 4, the highest corrosion current density was found in experiment 12, which was a combination of pH 6 and the highest chloride and sulfate concentrations of 1085.20 and 670.04 ppm, respectively, in the synthetic soil solution. Meanwhile, the lowest corrosion rate was obtained in experiment number 9, with a pH of 6 and the lowest chloride and sulfate concentrations of 85.20 and 70.04 ppm, respectively. Therefore, the results show that chloride and sulfate concentrations seem to be a negative influence, meaning that increases in these two factors will increase corrosion current density.

The input corrosion current density was then analyzed by RSM using Minitab 19 to obtain a mathematical model of the relationship of the research variables. Based on estimates of the parameters and experimental results, RSM revealed an empirical relationship between the response and the input variables, as expressed by the fitted regression model below. The regression second-order polynomial equation for corrosion current density developed using BBD is shown in the equation below in terms of uncoded factors,

*i*_corr_ (μA/cm^2^) = 5.840 − 0.542 · pH − 0.001217 · [Cl^−^] + 0.001539 · [SO_4_^2−^] + 0.0438 · [pH]^2^ + 0.000004 · [Cl^−^]^2^ − 0.000000 · [SO_4_^2−^]^2^ + 0.000050pH · [Cl^−^] − 0.00000 pH · [SO_4_^2−^] + 0.00000 [Cl^−^] · [SO_4_^2−^], (3)

The parameters [SO_4_^2−^]^2^, pH· [SO_4_^2−^], and [Cl^−^] · [SO_4_^2^^−^] in Equation (3) are independent of corrosion current density because their coefficients are zero. For the convenience of observation, insignificant parameters can be omitted and Equation (3) can be rewritten with uncoded factors, as follows,

*i*_corr_ (μA/cm^2^) = 5.840 − 0.542·pH − 0.001217·[Cl^−^] + 0.001539·[SO_4_^2−^] + 0.0438·[pH]^2^ + 0.000004·[Cl^−^]^2^ + 0.000050 pH·[Cl^−^], (4)

From Equation (4), the predicted results shown in Table 4 were calculated. In addition to the linear effect of the parameter for the corrosion current density of carbon steel in soil, Equation (4) also provided an understanding of the quadratic and interaction effects of the parameters. Verification of the equation’s reliability will be clarified in the following sections.

### 3.2. Validity Evaluation of the Fitted Model

Analysis of variance (ANOVA) was conducted to investigate the relationship between a response variable and one or more predictor variables [26].

This study used standard F_critical_ = F (9, 5, 0.05) = 4.7725, where 9 is the degree of freedom of regression, 5 is the degree of freedom of residual error, and 0.05 is the level of significance. Table 5 shows the model had a high calculated F-value of 812.85. The calculated F-value is much higher than F_critical_, indicating that it can reject the null hypothesis that all the coefficients are zero, and this model is statistically significant [27]. The F statistic must be used in combination with the *p*-value when deciding if the overall results are significant. The *p*-value is determined by the F statistic and is the probability that the results could have happened by chance. If the *p*-value is less than the alpha level, then the results are not significant and the null hypothesis cannot be rejected. In the sciences, the alpha level is usually set to be less than 0.05, which indicates model validation. Therefore, when the *p*-value output from the ANOVA of this study was 0.000, there was a significant difference between variables.

Another important value in ANOVA is lack of fit; the F-value for lack of fit with the null hypothesis that the model is an appropriate fit for the data. Therefore, in contrast to the F-value of the model, the null hypothesis should be “can reject”, F value of lack of fit with the null hypothesis should be “cannot be rejected”. In this study, the calculated F-value of lack of fit is 0.33 and the *p*-value is 0.808, with standard F_critical_ = F(3, 2, 0.05) = 19.1643, where 3 is the degree of freedom of lack of fit, 2 is the degree of freedom of pure error, 0.05 is the level of significance. In Table 5, F-value of lack of fit is much smaller than F_critical_; the *p*-value is higher than 0.05. Thus, the null hypothesis cannot be rejected, the relationship assumed in the model is reasonable, and there is no lack of fit in this model.

Moreover, the value of R^2^ is 99.93%, which indicates a good relationship between the experimental and predicted values of the response, with only 0.07% of the total variation not explained by the empirical model. The adjusted value (R^2^ adj) is 99.81%, which suggests that the total variation of 0.19% for corrosion current density can be attributed to the independent parameters. The closeness between R^2^ and adjusted R^2^ implies a high significance for the model. The analysis and observations revealed a strong correlation between the experiments results and the values predicted by the statistical model, demonstrating the model success.

### 3.3. Preliminary Study of the Effects of pH, Chloride, and Sulfate Concentrations on the Corrosion Current Density in a Soil Environment

**Figure 4:**
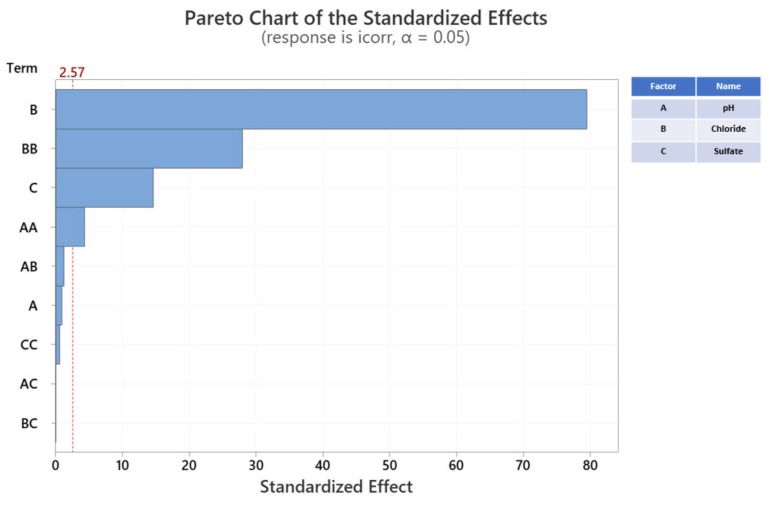
Pareto chart for the standardized effects of pH, chloride concentration, and sulfate concentration on the corrosion current density of carbon steel in a soil environment.

An F-test can show if a group of variables is jointly significant and a T-test can indicate if a variable is statistically significant. To precisely determine the effect of each factor on the corrosion current density (i.e., which influence is reliable and which is not), the Pareto chart in Figure 4 shows the standardized effects of pH, chloride, and sulfate concentrations on the corrosion current density evaluation. The T-value, which is used to measure how large the coefficient is in relationship to its standard error, is equal to the coded coefficient divided by its standard error. The Pareto chart was used to determine the magnitude and importance of the effects. Each bar represents the T-value for a type of factor; the height of the bar represents any important factors. Therefore, the biggest influence on the corrosion current density of carbon steel is chloride concentration. On the Pareto chart, bars that cross the reference line are statistically significant. In Figure 4, the bars that represent factors B, BB, C, and AA cross the reference line at 2.57. These factors are statistically significant at the 0.05 level in terms of the current model. Because the Pareto chart displays the absolute value of the effect, it determines large effects; however, it cannot determine effects that increase or decrease the response. A normal probability plot of the standardized effect is used to examine the magnitude and direction of the effects on one plot. In this case, all parameters have a significant influence on the corrosion current density when their *p*-value is less than 0.05. If the *p*-value is greater than 0.10, the model terms are insignificant. In this case, the *p*-value of pH is 0.403, [SO_4_^2−^] ·[SO_4_^2−^] is 0.562, pH·[Cl^−^] is 0.253, pH·[SO_4_^2−^] is 1.000 and [Cl^−^]·[SO_4_^2−^] is 1.000, which are inefficient model terms as shown in Table 6. However, the *p*-value of the model shown in Table 5 is 0.000; therefore, removing them is not necessary. The Pareto chart and Table 6 are in agreement with another study regarding separate factors. The correlation between pH and corrosion rates is very low in this model, and it was inconclusive regarding the effective correlation between pH and corrosion current density. There are other studies with different results on this issue [28,29,30]. Of the three factors investigated in this study, it can be concluded that the sulfate concentration effect is minor while the chloride concentration has a major influence on the corrosion rate of carbon steel in a soil environment.

### 3.4. Representation of Model: Response Surface Plotting and Contour Plot of Corrosion Current Density with Each Factor of Carbon Steel in the Soil Environment

Using Statistica software, two-dimensional (2D) surface contour plots and three-dimensional (3D) plots were generated to estimate the value of variation response to better understand the effect of each factor on the corrosion current density. Surface plots of the response function were useful for understanding both the individual and combined effects of pH and chloride. This study examined a combination of the effects of three different factors, but the 3D plots could only represent two factors and the response corrosion current density of carbon steel. Therefore, one factor must be given a fixed value (i.e., a hold value) to represent the other two factors.

### 3.5. Interactive Effect of pH and Chloride Concentration (ppm)

**Figure 5:**
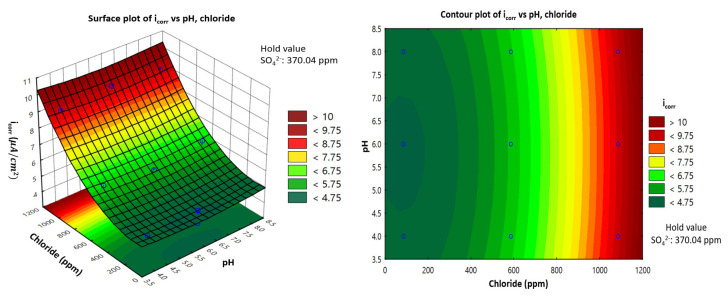
Response surface and contour plots showing the effect of pH and chloride concentration on the corrosion current density (*i*_corr_) of carbon steel in a soil environment.

The obtained results were represented using a 3D representation of the response surface plot and 2D contour plots. Figure 5 presents the 3D plots demonstrating the effects of pH and chloride on corrosion current density under predefined conditions. In Figure 5, with a hold value of 370.04 ppm for sulfate, the effect of chloride increased by a considerable amount for a corrosion current density from 4$\;\mu A/{\mathrm{cm}}^{2}$ to 9.2 $\mu A/{\mathrm{cm}}^{2}$. Meanwhile, the corrosion current density seemed to have very small variations in value (almost unchanging) as the pH value increased, which suggests that the corrosion current density is pH-independent.

### 3.6. Interactive Effect of pH and Sulfate Concentration (ppm)

**Figure 6:**
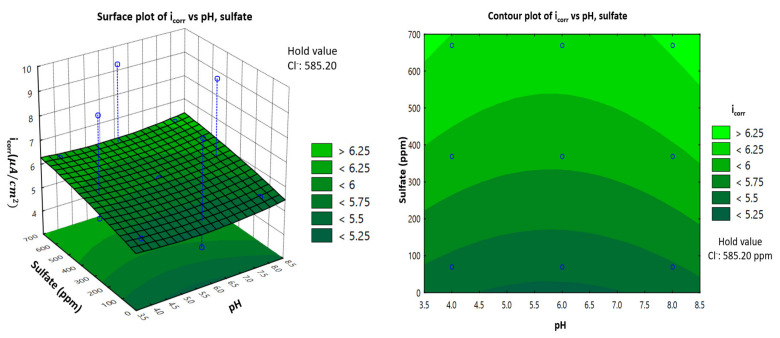
Response surface and contour plots showing the effect of pH and sulfate concentration on the corrosion current density (*i*_corr_) of carbon steel in a soil environment.

Figure 6 shows the response surface and contour plots for the effect of pH and sulfate on the corrosion current density. With a hold value of 585.02 ppm for chloride, the corrosion current density increased gradually when sulfate concentration increased, from 4 to 6.2 $\mu A/{\mathrm{cm}}^{2}$. However, with an increase in pH, there was a slight variation in corrosion current density, which was more significant than that in Figure 5. However, the pH-related variation was still considered to be negligible, and the pH seemed to be independent of the corrosion current density.

### 3.7. Interactive Effect of Chloride and Sulfate Concentrations (ppm)

**Figure 7:**
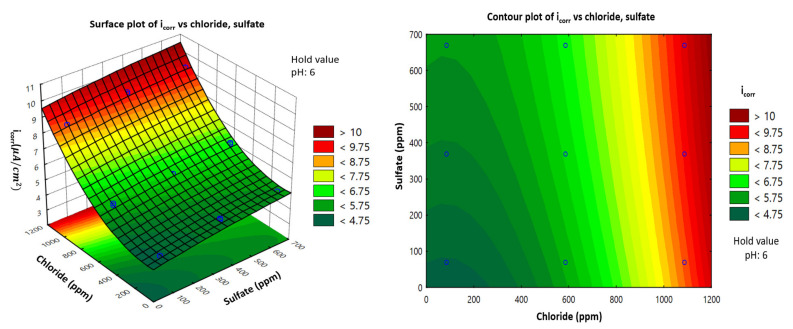
Response surface and contour plots showing the effect of chloride and sulfate concentrations on the corrosion current density (*i*_corr_) of carbon steel in a soil environment.

Figure 7 shows the effect of chloride and sulfate concentrations on the corrosion current density. The surface 2D contour plots and 3D response demonstrate the promotion efficiency. At a hold value of 6 for pH, an increased chloride or sulfate concentration was associated with an increased corrosion current density of carbon steel. From the minimum to maximum values for sulfate concentration, the corrosion current density increased from 4.2 to 5.5$\;\mu A/{\mathrm{cm}}^{2}$. Meanwhile, from the minimum value of chloride concentration to the maximum value of sulfate concentration, the corrosion current density increased from 4.2 to more than 9$\;\mu A/{\mathrm{cm}}^{2}$, which indicates quite a large influence. Several other studies have investigated this behavior and agree with these results [31,32,33].

## 4. Conclusions

Based on the results, the following conclusions could be drawn:

- The effects of pH, chloride concentration and sulfate concentration on the corrosion behavior of a carbon steel pipeline in a soil environment were investigated by statistical method RSM. Research results could be concluded that chloride and sulfate concentrations are a negative influence, pH seemed to be independent of the corrosion current density. A useful mathematical model was suggested for use in exploring methods to protect the buried pipeline.
- The effect level of independent variables on the corrosion rate was found to follow an increasing sequence of pH < sulfate concentration < chloride concentration.

The results show that the model was successful; however, it has a limitation because it can only be used within the experimental ranges. As chloride was the important factor influencing corrosion, further research with expanded chloride concentration ranges is necessary.

## Figures and Tables

**Table 1:** Initial concentrations and investigated value range of factors in synthetic soil solution [24].

Investigated Factor	pH	Chloride [Cl^−^], ppm	Sulfate [SO_4_^2−^], ppm
Initial value	6.8	85.2	70.04
Investigated value range	4–8	85.2–1085.2	70.04–670.04

**Table 2:** Actual investigated values of factors and their corresponding coded levels.

Variable	Code Values		
	−1 (Minimum)	0 (Medium)	1 (Maximum)
X_1,_ pH	4	6	8
X_2,_ Chloride (ppm)	85.20	585.20	1085.20
X_3,_ Sulfate (ppm)	70.04	370.04	670.04

**Table 3:** Experimental design based on the Box–Behnken Design matrix for the three variables.

Standard Run	Coded Parameter			Real Parameter		
	X_1_	X_2_	X_3_	pH	[Cl^−^], (ppm)	[SO_4_^2−^], (ppm)
1	−1	−1	0	4	85.2	370.04
2	1	−1	0	8	85.2	370.04
3	−1	1	0	4	1085.2	370.04
4	1	1	0	8	1085.2	370.04
5	−1	0	−1	4	585.2	70.04
6	1	0	−1	8	585.2	70.04
7	−1	0	1	4	585.2	670.04
8	1	0	1	8	585.2	670.04
9	0	−1	−1	6	85.2	70.04
10	0	1	−1	6	1085.2	70.04
11	0	−1	1	6	85.2	670.04
12	0	1	1	6	1085.2	670.04
13	0	0	0	6	585.2	370.04
14	0	0	0	6	585.2	370.04
15	0	0	0	6	585.2	370.04

**Table 4:** Experimental observations and predicted data for response variables obtained from the BBD.

Experiment Run	Factor			$\mathrm{Response}\;i_{\mathrm{corr}},\;\mu A/{\mathrm{cm}}^{2}$	
	pH	Chloride, ppm	Sulfate, ppm	Experiment Observation	Predicted
9	6	85.2	70.04	4.2	4.22
1	4	85.2	370.04	4.9	4.88
2	8	85.2	370.04	4.8	4.84
11	6	85.2	670.04	5	5.15
5	4	585.2	70.04	5.4	5.23
6	8	585.2	70.04	5.5	5.31
13	6	585.2	370.04	5.7	5.57
14	6	585.2	370.04	5.8	5.57
15	6	585.2	370.04	5.6	5.57
7	4	585.2	670.04	6.2	6.18
8	8	585.2	670.04	6.3	6.23
10	6	1085.2	70.04	8.6	7.99
3	4	1085.2	370.04	9.1	8.55
4	8	1085.2	370.04	9.2	8.7
12	6	1085.2	670.04	9.4	8.91

**Table 5:** Analysis of variance (ANOVA) for the fitted equation.

Source	Degree of Freedom	Adj. Sum of Square	Adj. Mean Square	F-Value	F_critical_	*p*-Value	Remarks
Model	9	43.8940	4.8771	812.85	4.7725	0.000	Significant
Error	5	0.0300	0.0060	-	-	-	-
Null hypothesis: All the coefficients are zero $\beta_{1}=\beta_{2}=\beta_{3}=\beta_{11}=\beta_{22}=\beta_{33}=\beta_{12}=\beta_{13}=\beta_{23}=0$							
Lack-of-Fit	3	0.0100	0.0033	0.33	19.1643	0.808	Reasonable
Pure Error	2	0.0200	0.0100	-	-	-	-
Total	14	43.5200	-	-	-	-	-
Null hypothesis: Model is an appropriate fit for the data→No lack of fit							
R^2^: 99.93%		R^2^ (adj.): 99.81%			R^2^ (pred.): 99.53%		

**Table 6:** Estimated coefficients with coded units for corrosion current density (*i*_corr_).

Term	Coefficient	Standard Error Coefficient	T for H_0_^a^ Coefficient = 0	*p*-Value
Constant	5.7000	0.0447	127.46	0.000
pH	0.0250	0.0274	0.91	0.403
[Cl^−^]	2.1750	0.0274	79.42	0.000
[SO_4_^2−^]	0.4000	0.0274	14.61	0.000
pH · pH	0.1750	0.0403	4.34	0.007
$[{\mathrm{Cl}}^{-}]\;\cdot [$Cl^−^]	1.1250	0.0403	27.91	0.000
$[{\mathrm{SO}}_{4}2^{-}]\;\cdot$ [SO_4_^2−^]	−0.0250	0.0403	−0.62	0.562
pH ·[Cl^−^]	0.0500	0.0387	1.29	0.253
pH ·[SO_4_^2−^]	0.0000	0.0387	0.00	1.000
$[{\mathrm{Cl}}^{-}]\;\cdot [$SO_4_^2−^]	0.0000	0.0387	0.00	1.000

## Data Availability

Data sharing is not applicable to this article.
